# Genome-wide statistical evidence elucidates candidate factors of life expectancy in dogs

**DOI:** 10.1016/j.mocell.2024.100162

**Published:** 2024-11-22

**Authors:** Won Hee Ko, Sangil Kim, Alix Catry, Je-Yoel Cho, Seunggwan Shin

**Affiliations:** 1 School of Biological Sciences and Institute of Biodiversity, Seoul National University, Seoul 08826, Republic of Korea; 2 Comparative Medicine Disease Research Center, Seoul National University, Seoul 08826, Republic of Korea; 3 Museum of Comparative Zoology and Department of Organismic and Evolutionary Biology, Harvard University, Cambridge, MA 02138, USA; 4 Université Claude Bernard Lyon 1, Laboratoire d'Ecologie Microbienne, UMR CNRS 5557, UMR INRAE 1418, VetAgro Sup, 69622, Villeurbanne, France; 5 Department of Biochemistry, BK21 Plus and Research Institute for Veterinary Science, College of Veterinary Medicine, Seoul National University, Seoul 08826, Republic of Korea

**Keywords:** *Canis lupus familiaris*, Comparative genomics, Dog breeds, Genome-wide association study, Lifespan

## Abstract

It is well-established that large and heavy dogs tend to live shorter lives. In this study, we aimed to determine whether traits other than body size are associated with the life expectancy of dogs. We compiled a dataset of 20 phenotypes, including body size, lifespan, snout ratio, and shedding, into a single matrix for 149 dog breeds using data from the American Kennel Club and other peer-reviewed sources. The analysis revealed that drooling might be associated with both the lifespan and body mass index of dogs. Furthermore, a genome-wide association study with adjusted phenotypes and statistical verification methods, such as Mendelian randomization. Additionally, conducting differential gene expression analysis with the salivary gland for the 2 cases, hypersalivation/less drooling vs various body sizes, we could observe the hypersalivation-related proteins. This genetic analysis suggests that body size and drooling might be candidate factors influencing lifespan. Consequently, we identified several candidate genes, including *IGSF1*, *PACSIN2*, *PIK3R1*, and *MCCC2*, as potential genetic factors influencing longevity-related phenotypes.

## INTRODUCTION

Domestic dogs, *Canis lupus familiaris*, are the most phenotypically variable mammals, exhibiting significant variation in size, shape, color, behavior, disease risk, and lifespan. Larger breeds typically live shorter lives (6-7 years) compared to smaller breeds, which can live up to 15 years, with aging increasing disease and mortality risk (Kraus et al., 2013). This diversity among breeds provides a valuable model for studying genetic and environmental factors influencing aging, offering insights applicable to all mammals, including humans (Fleming et al., 2011, Lee and Lee, 2022, Ruple et al., 2022, Wayne and Ostrander, 2007). Studies with dogs enhance our understanding of aging and age-related diseases, complementing laboratory models by investigating aging in real-world conditions (O'Neill et al., 2014).

Genetic studies have identified insulin-like growth factor 1 (*IGF1*) as a key gene related to body size and lifespan in dogs, with parallels in humans (Berryman et al., 2008, Greer et al., 2011, Sutter, 2007, Ziv and Hu, 2011). Experimental evidence shows that *IGF1* manipulation in animal models affects both body size and lifespan, underscoring its role in these processes (Lorenzini et al., 2014, Yakar and Adamo, 2012). However, longevity is a polygenic and complex trait influenced by multiple genetic and environmental factors (López-Otin et al., 2023). Breeds, such as French Bulldogs and Boxers, affected by breed-specific health issues due to artificial selection, often have reduced life expectancy, particularly in brachycephalic breeds prone to disorders such as ptyalism and kidney failure (Bannasch et al., 2021, Lodato and Hedlund, 2012, Mitze et al., 2022, O’Neill et al., 2018). Thus, factors beyond body size also contribute to dog's lifespan.

We collected data on 149 dog breeds, covering approximately 75% of all American Kennel Club registered breeds, and compiled it into a data matrix for statistical studies such as comparative genomics. Using this matrix, we conducted statistical analyses to identify phenotypic traits associated with life expectancy. We performed genome-wide association study (GWAS) analyses using a previously published single nucleotide polymorphism (SNP) dataset of 722 individuals, including wild canines and village dogs (Plassais et al., 2019). This analysis revealed both known loci associated with life expectancy and new genic regions with potential candidate genes. Through Mendelian randomization (MR) and differential gene expression (DEG) analyses, we identified several relevant genes, including *IGSF1*, protein kinase C and casein kinase substrate in neurons 2 (*PACSIN2*), *PIK3R1*, and *MCCC2*.

## MATERIALS AND METHODS

### Phenotype Matrix and Statistical Analysis

We compiled 20 phenotypes for 149 dog breeds, encompassing 722 datasets, using American Kennel Club, Fédération Cynologique Internationale, and peer-reviewed sources (Hayward et al., 2016, Jones et al., 2008). Since the dataset lacked individual phenotype or disease records and adhered to strict breed standards, morphological traits were defined as breed averages, following methods used in previous studies (Hayward et al., 2016, MacLean et al., 2019, Spady and Ostrander, 2008). The phenotypes were organized into a matrix, with each breed assigned a value (Appendices S1 and S2). Correlation analyses between lifespan and other phenotypes were conducted using R (R core team, 2023). We removed non-numeric variables, converted basal and nonbasal breeds to numeric values, and eliminated missing data using tidyverse (Wickham, 2019), checking for linearity and collinearity (Fig. S1). Pearson correlation was used for nominal datasets (eg, lifespan and body mass index [BMI]), while polyserial correlation was applied to nominal and ordinal datasets (eg, lifespan and drooling) using the polycor (https://r-forge.r-project.org/projects/polycor) package in R. Breeds were categorized into small, medium, and large body sizes for grouped correlation analysis.

### GWAS and MR Analysis

We utilized the 722 datasets by converting the original variant call format to a PLINK binary file format (Purcell et al., 2007), removing insertions/deletions and multiallelic variants to focus on biallelic SNPs. After filtering with a 0.05 minor allele frequency and 0.01 missingness threshold, we obtained approximately 8.1M SNPs. Phenotype adjustments for drooling, sex, and body size were conducted using a linear regression model in R. We then performed a GWAS using GEMMA v0.98.3 (Zhou and Stephens, 2012) on 3 phenotypes: drooling, BMI, and lifespan, employing a univariate linear mixed model and the Wald test for *P*s. Significant associations were visualized with a multitrack Manhattan plot using the CMplot package (Yin et al., 2021) in R. For MR analysis, we used the TwoSampleMR R package (Hemani et al., 2017). Linkage disequilibrium clumping was conducted with PLINK (*r*^2^ threshold as 0.01), and genome-wide significant SNPs were selected for instrumental variance. We harmonized effect sizes for BMI and drooling with lifespan-associated SNPs and performed MR analysis using 5 regression models, visualized with forest and funnel plots. Sensitivity analysis was also conducted using the mr_singlesnp and mr_forest_plot functions in the TwoSampleMR package.

### DEG Analysis

To investigate additional candidate genes, we downloaded breed-specific salivary gland RNA-seq data from National Center for Biotechnology Information (NCBI) (PRJNA396033) for 3 breeds: Belgian Malinois (SRR8997026), Yorkshire Terrier (SRR8997053), and Newfoundland (SRR8996972). We used Trinity v2.14.0 (Grabherr et al., 2011) for de novo transcriptome assembly, incorporating Trimmomatic for adapter trimming. Quality was assessed using BUSCO v5.4.2 (Simao et al., 2015) against the carnivora_odb10 database, and redundant sequences were removed with CD-hit-est (Fu et al., 2012). We quantified gene expression with RNA-seq by Expectation-Maximization (RSEM) (Li and Dewey, 2011), aligning reads with Bowtie2 (Langmead and Salzberg, 2012), and conducted DEG analysis with Trinity’s run_DE_analysis.pl, visualizing results with R. Significant DEGs were identified with a *P* cutoff of 1E-03 and log(FC) >1.5. For functional annotation, we used TransDecoder (github.com/TransDecoder/TransDecoder) to predict open reading frames and Trinotate (Bryant et al., 2017) to integrate annotation data, including BLASTP, BLASTX (Camacho et al., 2009), HMMER (Eddy, 2008), and signalP5 (Armenteros et al., 2019) results. We created a comprehensive annotation report in Excel, including KEGG pathway information (Kanehisa and Goto, 2000). Gene ontology term analysis was performed using topGO (Alexa et al., 2006; Ashburner et al., 2000) to visualize breed-specific enriched subsets.

## RESULTS

### Analysis of Correlation Between Phenotype Matrix Using Association Test

We gathered data on 149 dog breeds from the 722 datasets to explore phenotype-genotype associations through GWAS and statistical analyses. This comprehensive dataset, comprising 20 phenotypes (Appendix S1), enabled us to investigate lifespan-related factors. Missing data were minimal for physical traits such as weight and BMI (<1%) but higher for traits such as snout angle and coat colors (>30%) (Fig. S2). Correlation analysis revealed a strong negative correlation between lifespan and drooling (correlation coefficient = −0.81, *P* < 2.2E-16) and a positive correlation between drooling and BMI (correlation coefficient = 0.91, *P* < 2.2E-16) (Figs. S2 and S3). To address potential confounding effects, we analyzed drooling and lifespan correlations across 3 body size subgroups: x-small + small (correlation coefficient = −0.39, *P* = 1.4E-03), medium (correlation coefficient = −0.28, *P* = 1.7E-02), and large + x-large (correlation coefficient = −0.51, *P* = 4.087E-06), finding significant negative correlations in all groups. These results suggest that drooling may influence life expectancy independent of body size (Appendix S1). Before GWAS analysis, we adjusted for potential confounders such as sex and body size to reduce correlation bias (Figs. S2-S4). On the other hand, drooling may also be influenced by breed-specific health traits, such as brachycephalic syndrome, due to the lack of detailed information on the cephalic index for each breed, it was difficult to account for this confounding factor accurately.

### Identification of Candidate Genes Associated With Lifespan by GWAS

**Fig. 1:**
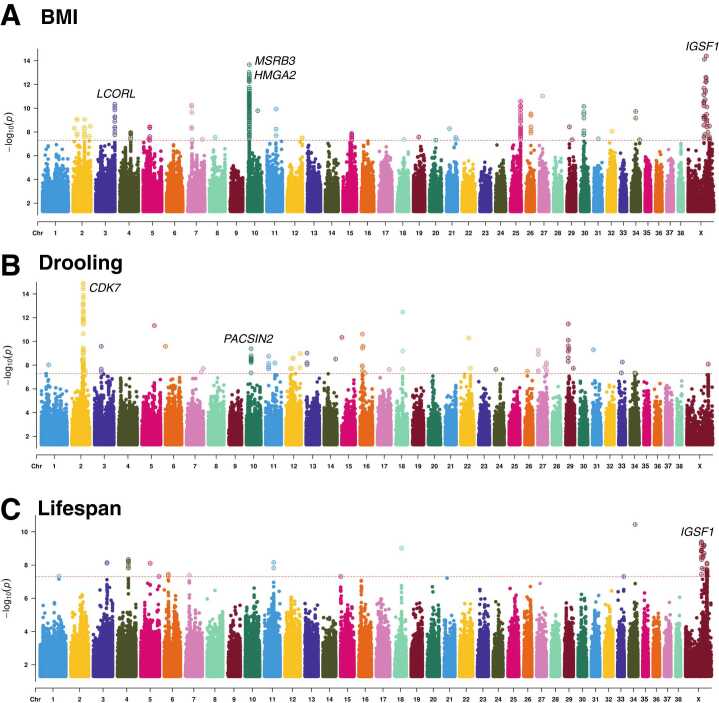
The multitrack Manhattan plot based on the GWAS results. (A) Manhattan plot displaying the GWAS results for body mass index (BMI) in dogs. The x-axis represents the chromosomal position, while the y-axis represents the −log10(*P*) of the SNPs. Significant loci identified include *MSRB3* and *HMGA2* on chromosome 10, *LCORL* on chromosome 3, and *IGSF1* on the X chromosome. (B) Manhattan plot displaying the GWAS results for drooling in dogs. The x-axis represents the chromosomal position, while the y-axis represents the −log10(*P*) of the SNPs. Significant loci identified include *CDK7* on chromosome 2, *PACSIN2* on chromosome 10, and *IGSF1* on the X chromosome. (C) Manhattan plot displaying the GWAS results for lifespan in dogs. The x-axis represents the chromosomal position, while the y-axis represents the −log10(*P*) of the SNPs. The significant locus identified is *IGSF1* on the X chromosome. Dashed horizontal lines indicate the genome-wide significance threshold. SNPs above this threshold are considered to be significantly associated with the trait of interest.

The GWAS was applied to the 3 phenotypes, including lifespan and potential candidate factors (BMI and drooling), fitting a linear mixed model to account for population structure. To avoid detecting false positives, we used the cutoff with the genome-wide significant threshold (5E-08) to identify significant associations in GWAS (Kaler and Purcell, 2019) (Fig. 1 and Table S1). All quantile-quantile plots and the inflation factors are represented in the Supplementary Information (Fig. S5 and Table S1). For BMI, our results align with previous research findings, specifically identifying methionine sulfoxide reductase B3 (*MSRB3*), high mobility group AT-Hook 2 (*HMGA2*), *IGF1*, and immunoglobulin superfamily member 1 (*IGSF1*) (Table S2). Regarding drooling, several genes were detected (Table S3), including cyclin-dependent kinase 7 (*CDK7*), with the highest peak observed on chromosome 2. Additionally, *PACSIN2* and aspartate beta-hydroxylase (*ASPH*) showed other peaks and are involved in critical cellular processes related to protein modification and membrane dynamics, respectively. Also, *IGSF1* in chromosome X was detected. Last, for lifespan, we detected *IGSF1* and rho GTPase activating protein 36 (*ARHGAP36*) on the X chromosome, which has been mentioned in previous studies. In addition, SNP rs24859997, was detected in the GWAS of the 3 phenotypes located upstream of *IGSF1*.

### Investigation of the Relationship Between Lifespan and Other Factors

**Fig. 2:**
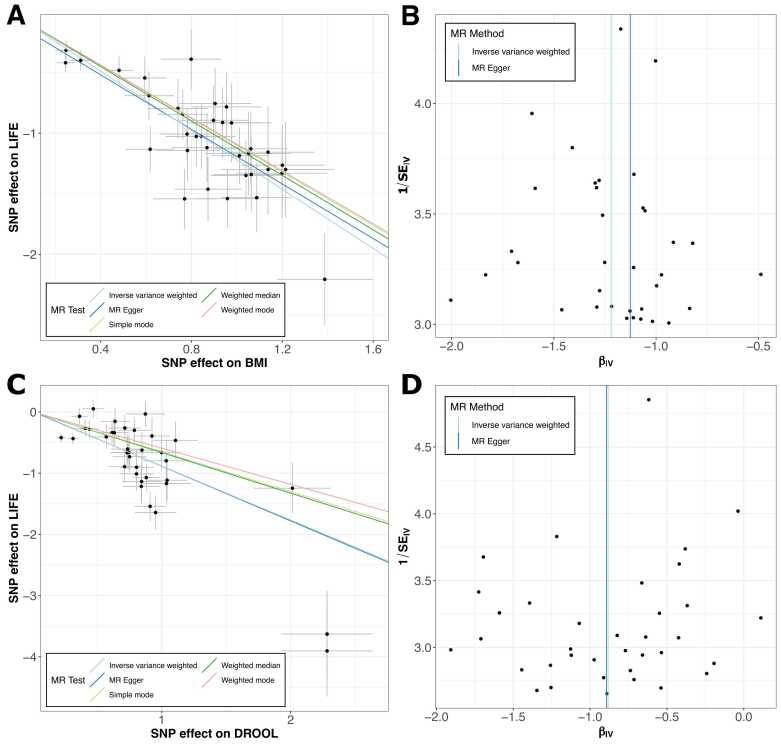
One-sample MR test results for BMI, drooling, and lifespan. (A) Scatter plot shows the effect of SNPs on BMI against their effect on lifespan. Each point represents an SNP, with error bars indicating standard errors. The plot includes lines from different MR methods: inverse variance weighted (IVW), MR Egger, weighted median, simple mode, and weighted mode. The symmetric pattern and the consistency across methods suggest a robust relationship. (B) Funnel plot assessing the presence of bias for the effect of BMI on lifespan. Each point represents an SNP. The symmetrical inverted funnel shape, with the MR Egger intercept near 0, indicates unbiased estimates and no significant directional pleiotropy. (C) Scatter plot shows the effect of SNPs on drooling against their effect on lifespan. Each point represents an SNP, with error bars indicating standard errors. The plot includes lines from different MR methods: IVW, MR Egger, weighted median, simple mode, and weighted mode. The symmetry and consistency across methods support the robustness of the relationship inference. (D) Funnel plot assessing the presence of bias for the effect of drooling on lifespan. Each point represents an SNP. The symmetrical inverted funnel shape, with the MR Egger intercept near 0, suggests unbiased estimates and no significant directional pleiotropy.

To investigate the relationship between lifespan (outcome) and drooling/BMI (exposures), we performed a 1-sample univariate MR analysis using genetic variants as instrumental variables (Reay and Cairns, 2021). The analysis utilized GWAS statistics for BMI and drooling against lifespan, focusing on SNPs with genome-wide significance and using linkage disequilibrium clumping for independence. We tested 5 MR models: MR Egger, weighted median, inverse variance weighted, simple mode, and weighted mode. Scatter and forest plots of SNP effects on BMI-lifespan and drooling-lifespan revealed significant relationships (Fig. 2). In the MR test, we observed a symmetric pattern—for the IVW method, the scatter of points formed a symmetrical inverted funnel shape—indicating that the instrumental variable estimates are unbiased. In the forest plots obtained from the single SNP analysis (Fig. S6), for BMI-lifespan and drooling-lifespan, rs24859997 (located upstream of *IGSF1*) showed a high MR effect size value (Tables S4 and S5). In addition, the pleiotropy test results indicate that for BMI-lifespan, the *P* is .3, and for drooling-lifespan, the *P* is .95. These indicate that the intercepts are not significantly different from zero, suggesting that there is no significant pleiotropy in either case. We detected a valid signal from the MR test using 2 genotype datasets, confirming a significant relationship not only between BMI and lifespan but also, between drooling and lifespan.

### Investigation of Gene Expression Differences in Salivary Glands Among Various Breeds

We conducted a DEG analysis using salivary gland RNA-Seq datasets to explore the relationship between drooling, BMI, and lifespan across dog breeds. We categorized our analysis into 3 groups: (1) different body size with differing drooling levels (Newfoundland vs Yorkshire Terrier), (2) similar body size with differing drooling levels (Newfoundland vs Belgian Malinois), and (3) different body size with the same drooling level (Belgian Malinois vs Yorkshire Terrier) (Appendix S3). BUSCO scores for the RNA-Seq data were 69.4%, 67.2%, and 70.1% for these comparisons, respectively (Table S6). RSEM results revealed that larger body size breeds commonly expressed higher levels of immunoglobulin proteins (Fig. S7, Tables S7, S8). In the Newfoundland vs Belgian Malinois comparison, Newfoundland, a hypersalivation breed, showed high *SCGB1A1* expression associated with hypersalivation (Côté et al., 2012). However, no hypersalivation-related genes were detected in comparing breeds with the same drooling level but different body sizes (Table S7). Gene ontology enrichment analysis showed that larger breeds upregulated genes related to immune responses, antigen binding, and the immunoglobulin complex (Table S8). Additionally, Newfoundland upregulated pathways related to metabolism and secretory granules, while Belgian Malinois upregulated pathways related to sensory perception of smell in both comparisons (Table S8).

## DISCUSSION

In this study, we compiled phenotype information for 149 dog breeds to explore traits associated with drooling and lifespan. Adjusting for sex and body size was crucial to isolate the genetic influences on these traits (Austad and Fischer, 2016, Uffelmann et al., 2021). Our GWAS identified key genes linked to drooling, including *CDK7*, *ASPH*, *PACSIN2*, *PIK3R1*, *MCCC2*, and *IGSF1*. *PACSIN2* is involved in gland function (Rual et al., 2005), *PIK3R1* is part of the PI3K/Akt pathway associated with longevity, *MCCC2* is related to metabolism (Zolotukhin, 2013), and *IGSF1*, located on the X chromosome, influences hormone secretion (Xue et al., 2005). These findings highlight the complex genetic and metabolic factors influencing drooling and its potential impact on longevity. Our combined GWAS and MR analyses suggest that drooling may be a lifespan-related phenotype. One-sample MR tests showed consistent and unbiased SNP effect estimates, with IVW and MR Egger plots indicating no significant directional pleiotropy (Burgess and Thompson, 2017, Richmond and Smith, 2022). SNP 15:41234395 on the *IGF1* gene showed a moderate association with BMI and lifespan, while SNP rs24859997 had a more significant effect on BMI, drooling, and lifespan, suggesting *IGSF1*'s key role in these traits (Joustra et al., 2020).

We conducted a DEG analysis of the salivary gland, comparing dogs with hypersalivation to those with less drooling while accounting for body size as a confounding factor, focusing on 3 breeds to examine groups with varying levels of BMI and drooling (Chibly et al., 2022). High-drooling breeds showed higher expression of genes related to salivary secretion, particularly *DMBT1*, which is upregulated in Newfoundlands and linked to immune response, epithelial cell differentiation, and tumor suppression (Mollenhauer et al., 2000). *SLPI*, coexpressed with *DMBT1* (coexpression score 0.078 from the GEO database, STRING), modulates inflammatory responses after bacterial infection, while *OLFMA4* and *SCGB1A1* are also associated with salivary gland function (Xu et al., 2020). Upregulated processes in Newfoundlands include the “hypochlorous acid metabolic process” and “secretory granule lumen,” both crucial for saliva secretion (Carvalho et al., 2019, Denny et al., 1997, Melvin et al., 2005, Turner and Sugiya, 2002). In Belgian Malinois, the “sensory perception of smell” pathway is upregulated, though it primarily influences appetite rather than hypersalivation (Carreira et al., 2020, Liu et al., 2021). Larger breeds showed higher expression of immunoglobulin proteins, possibly reflecting the impact of body size on immune response (Ruhs et al., 2020, Saitou et al., 2020). Our analysis highlights genes directly related to hypersalivation and suggests body size may explain some observed differences.

In summary, our study suggests that BMI and drooling could be the factors influencing the life expectancy of dogs. Our statistical tests and MR analysis indicate a possible relationship among these 3 phenotypes, warranting further investigation of the identified genes. Canine morphological diversity, shaped by domestication and selective breeding, impacts lifespan. Breeds known for hypersalivation, such as Newfoundland, Bloodhound, and Mastiffs, have shorter lifespans (8.8 years) compared to other large breeds (9.3 years) (Parker et al., 2017). Excessive drooling can lead to health issues such as kidney failure (Haley et al., 2011), potentially affecting overall health and longevity (Freiche and German, 2021). Further research on the link between hypersalivation and lifespan is recommended, considering both canine and comparative medicine.

## Author Contributions

W.H.K., S.K., S.S., and J.Y.C. conceived the project; W.H.K. performed analyses; W.H.K. wrote the manuscript and prepared figures; and A.C. compiled a dispersed phenotype dataset and calculated a correlation coefficient. J.Y.C., S.K., and S.S. revised the manuscript. All authors approved the submission of the final manuscript. Further information and requests for resources and reagents should be directed to and will be fulfilled by the lead contact, Seunggwan Shin (sk83 @snu.ac.kr).

## Declaration of Competing Interests

None.

## Footnotes

Appendix A Supplemental material associated with this article can be found online at: doi:10.1016/j.mocell.2024.100162.
