# Supplementary material for: Genome-wide statistical evidence elucidates candidate factors of life expectancy in dogs
Source: Mol Cells. 2024 Nov 22;48(1):100162. doi: 10.1016/j.mocell.2024.100162 (PMC11721540; doi:10.1016/j.mocell.2024.100162)
Supplement: Supplementary file 2 — Supplementary material [file mmc2.pdf]

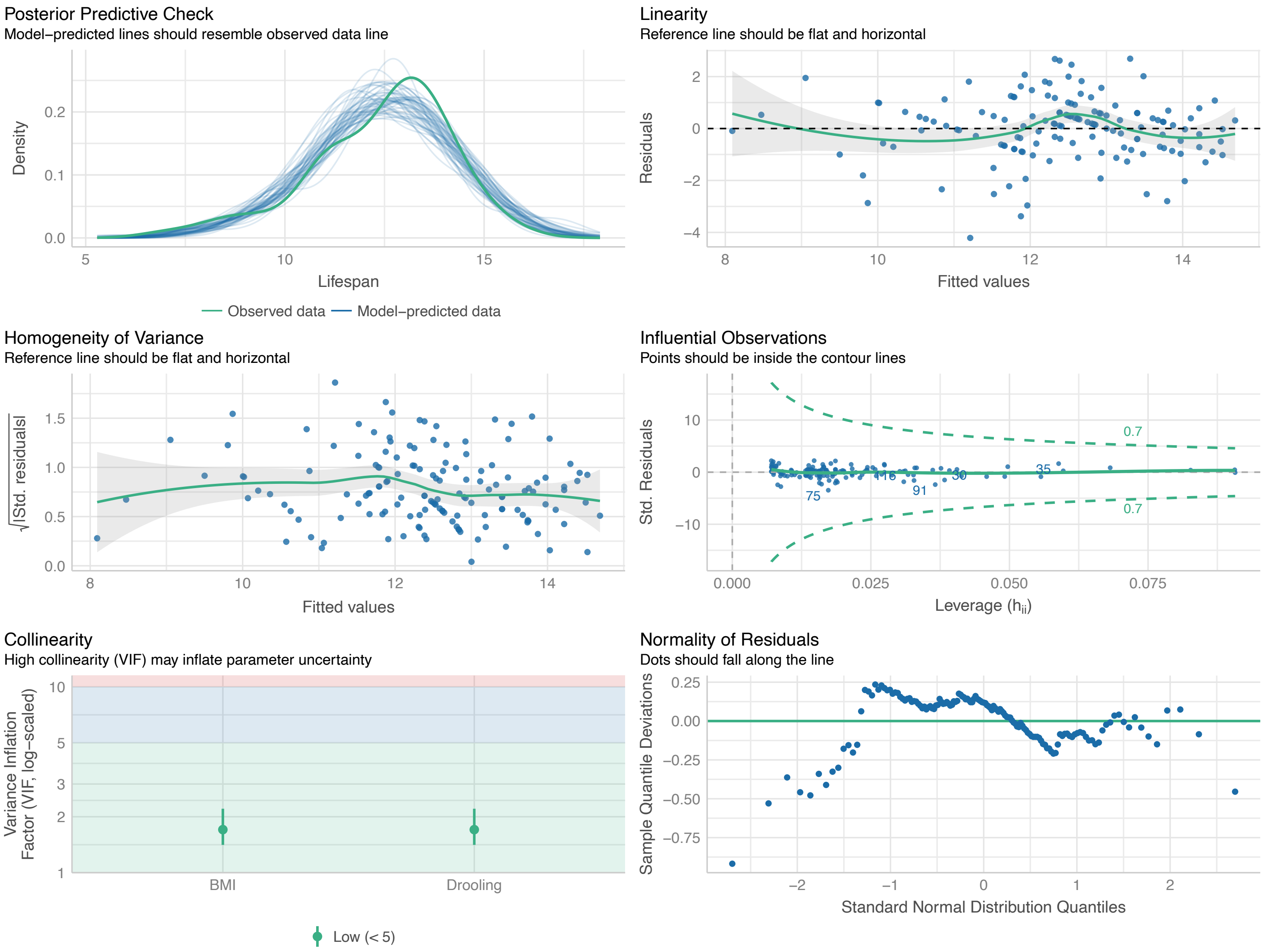

Posterior Predictive Check
Model−predicted lines should resemble observed data line
Density
0.0
0.1
0.2
5
10
15
Lifespan
Observed data
Model−predicted data
Linearity
Reference line should be flat and horizontal
Residuals
−4
−2
0
2
8
10
12
14
Fitted values
Homogeneity of Variance
Reference line should be flat and horizontal
√|Std. residuals|
0.0
0.5
1.0
1.5
8
10
12
14
Fitted values
Influential Observations
Points should be inside the contour lines
Std. Residuals
−10
0
10
0.7
0.7
75
116
91
30
35
0.000
0.025
0.050
0.075
Leverage (h_{ii})
Collinearity
High collinearity (VIF) may inflate parameter uncertainty
Variance Inflation Factor (VIF, log−scaled)
1
2
3
5
10
BMI
Drooling
Low (< 5)
Normality of Residuals
Dots should fall along the line
Sample Quantile Deviations
−0.75
−0.50
−0.25
0.00
0.25
−2
−1
0
1
2
Standard Normal Distribution Quantiles
