# Supplementary material for: Genome-wide statistical evidence elucidates candidate factors of life expectancy in dogs
Source: Mol Cells. 2024 Nov 22;48(1):100162. doi: 10.1016/j.mocell.2024.100162 (PMC11721540; doi:10.1016/j.mocell.2024.100162)
Supplement: Supplementary file 3 — Supplementary material [file mmc3.pdf]

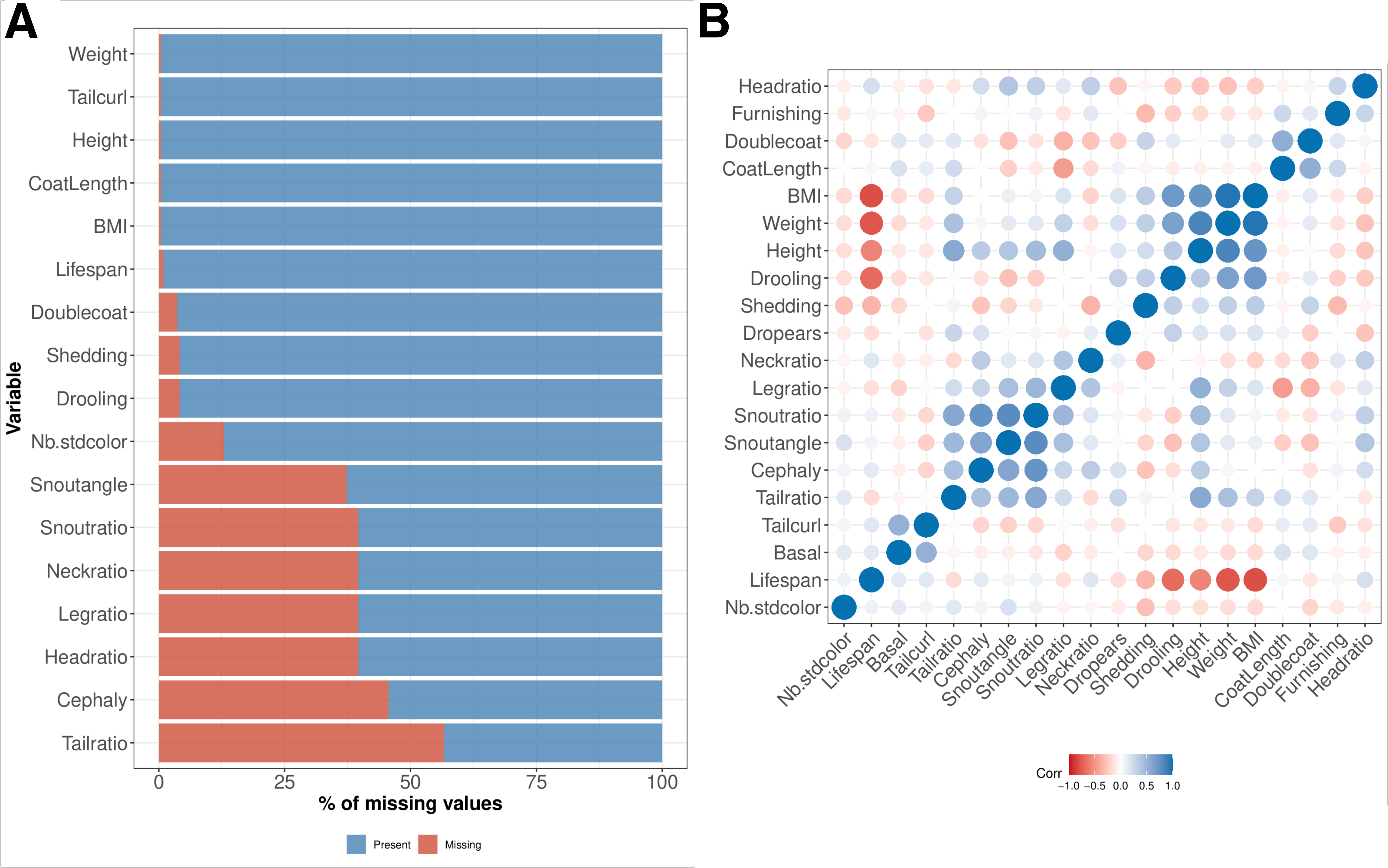

A
Weight
Tailcurl
Height
CoatLength
BMI
Lifespan
Doublecoat
Shedding
Drooling
Nb.stdcolor
Snoutangle
Snoutratio
Neckratio
Legratio
Headratio
Cephaly
Tailratio
Variable
0
25
50
75
100
% of missing values
Present
Missing
B
Headratio
Furnishing
Doublecoat
CoatLength
BMI
Weight
Height
Drooling
Shedding
Dropears
Neckratio
Legratio
Snoutratio
Snoutangle
Cephaly
Tailratio
Tailcurl
Basal
Lifespan
Nb.stdcolor
Corr
−1.0
−0.5
0.0
0.5
1.0
