# Supplementary figures and images for: Genome-wide statistical evidence elucidates candidate factors of life expectancy in dogs
Source: Mol Cells. 2024 Nov 22;48(1):100162. doi: 10.1016/j.mocell.2024.100162 (PMC11721540; doi:10.1016/j.mocell.2024.100162)

**A** Polyserial Correlation : 0.9059153

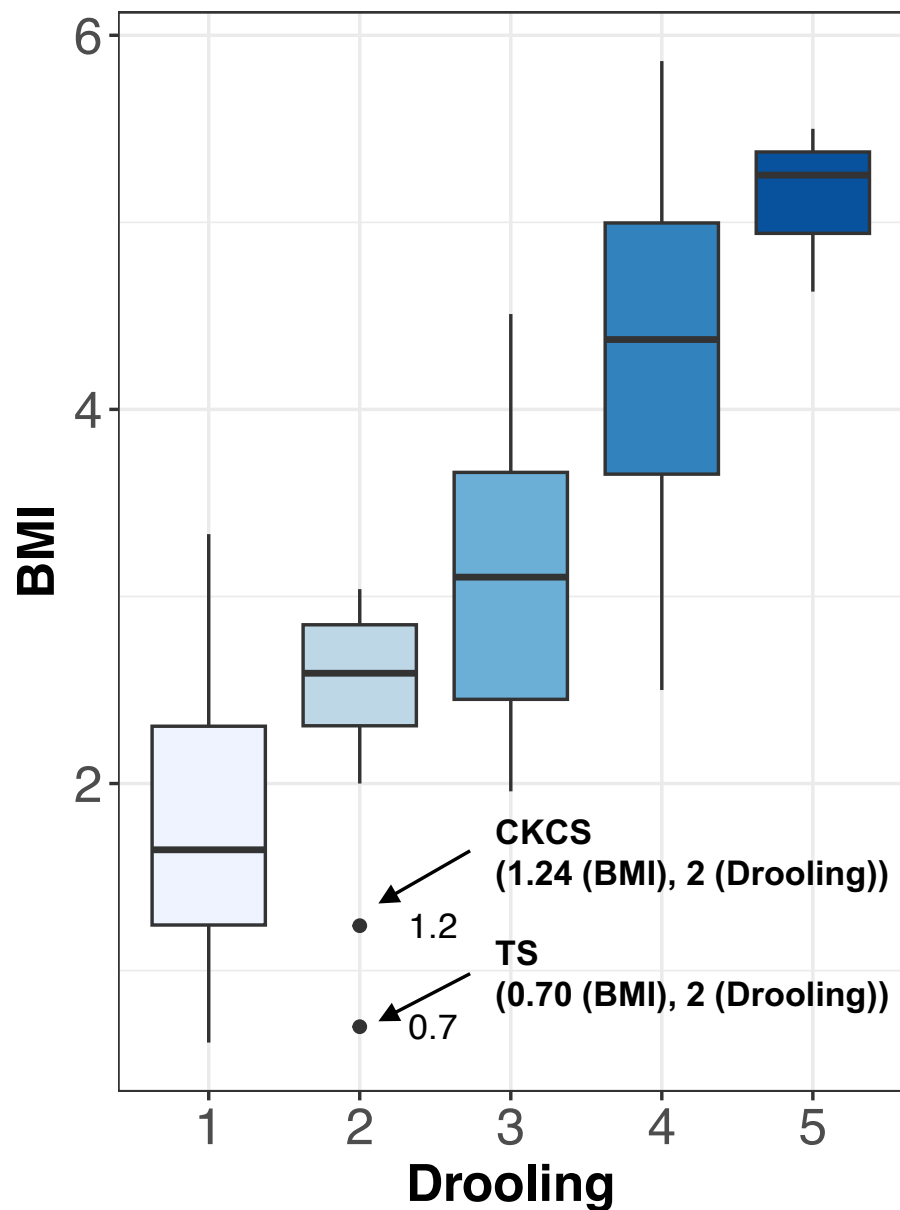

**B** Polyserial Correlation : -0.8146577

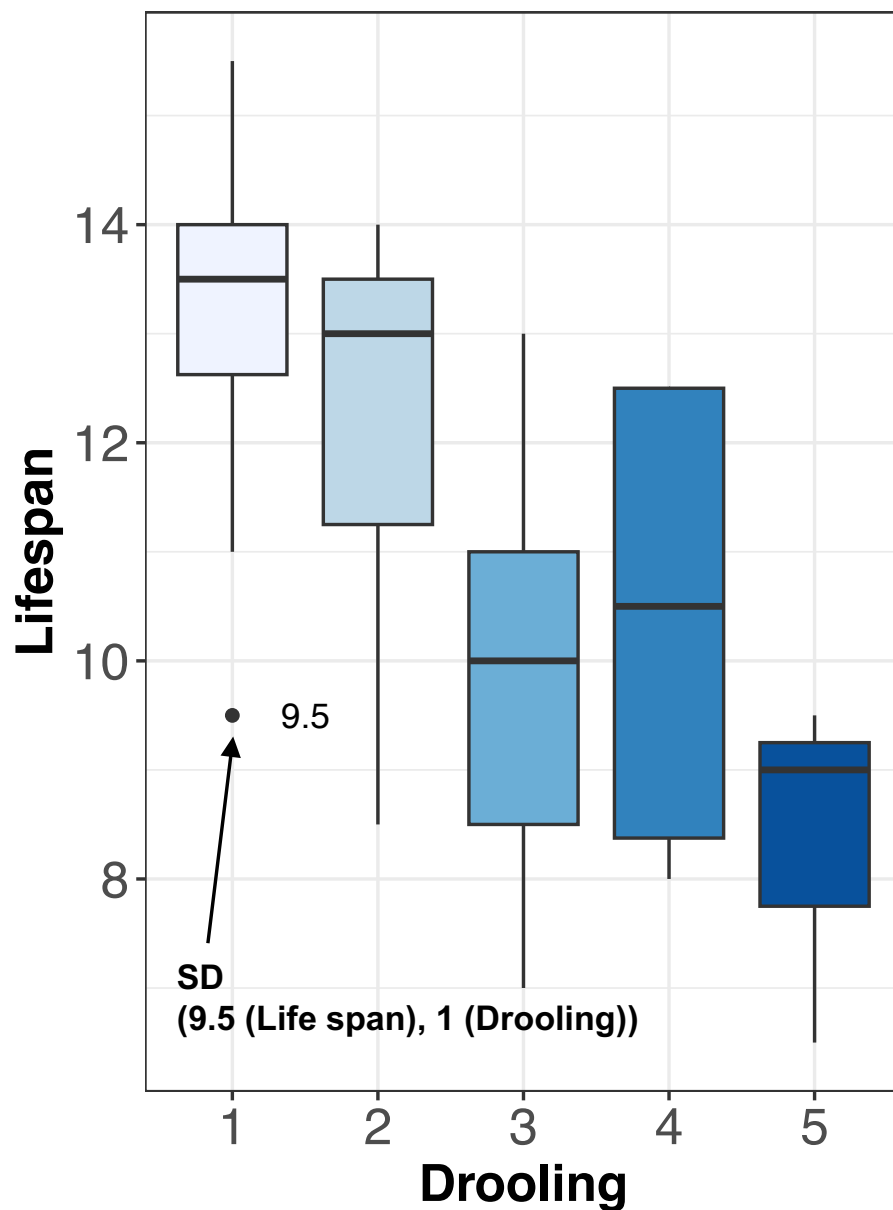

Supplement: Supplementary file 4 — Supplementary material [file mmc4.pdf]

**A** Original Phenotype Distribution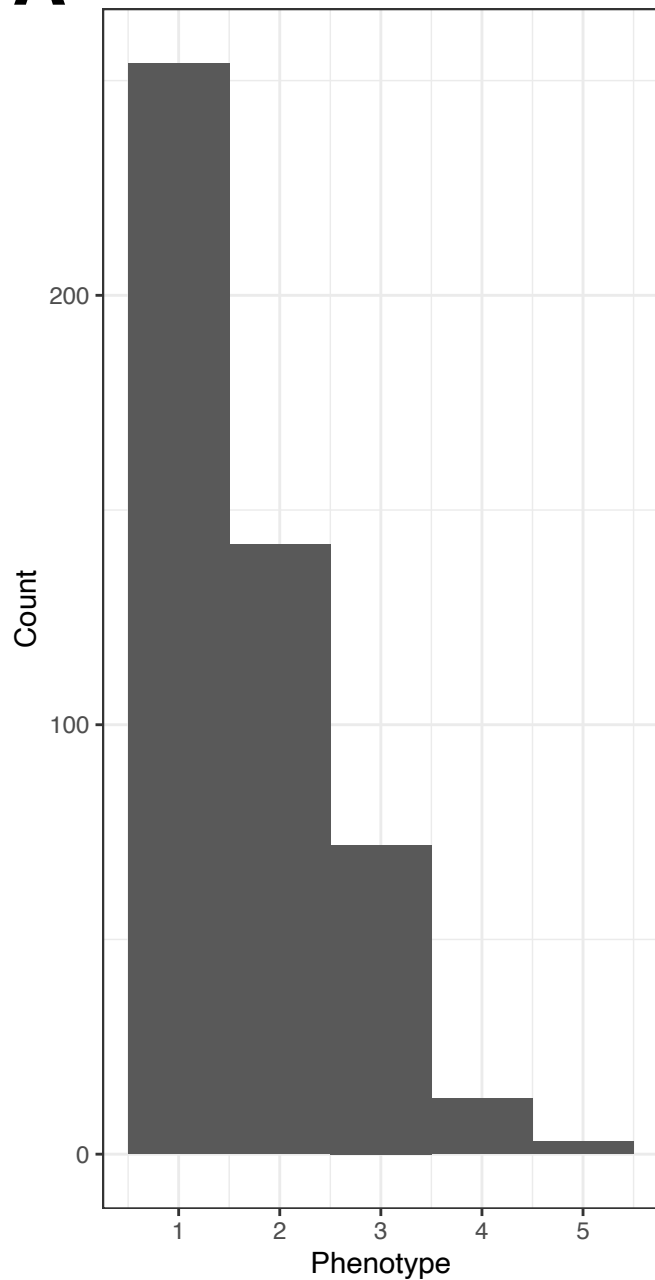**B** Adjusted Phenotype Distribution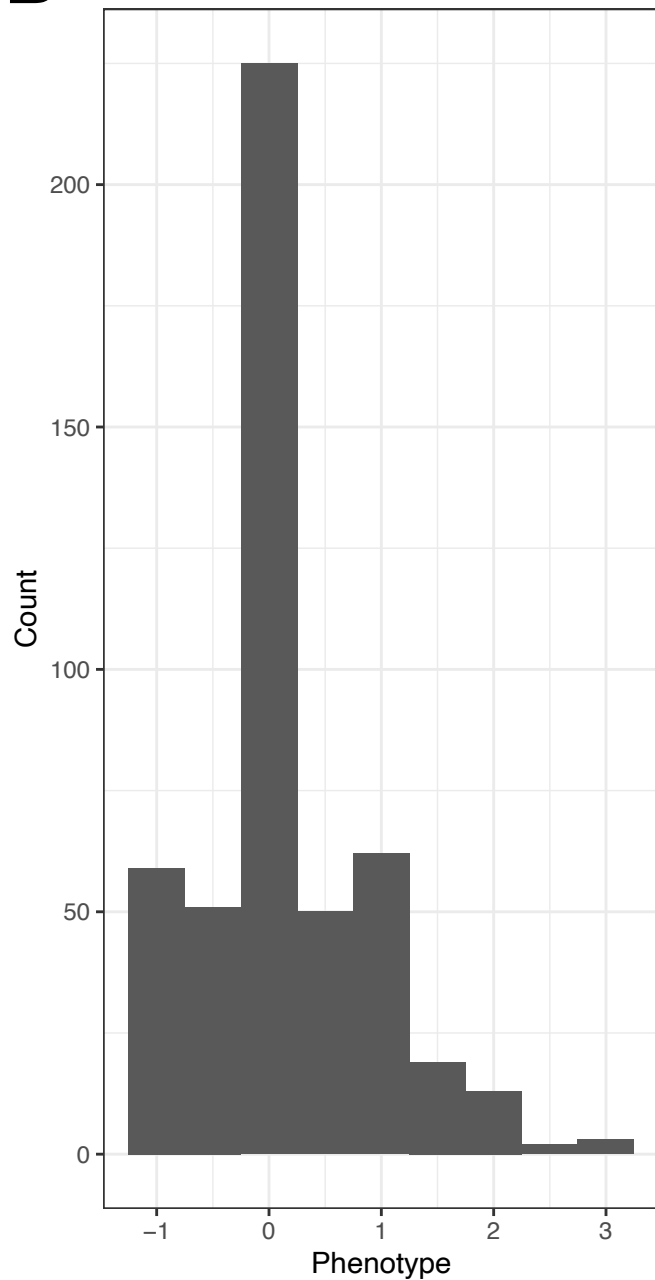

Supplement: Supplementary file 5 — Supplementary material [file mmc5.pdf]

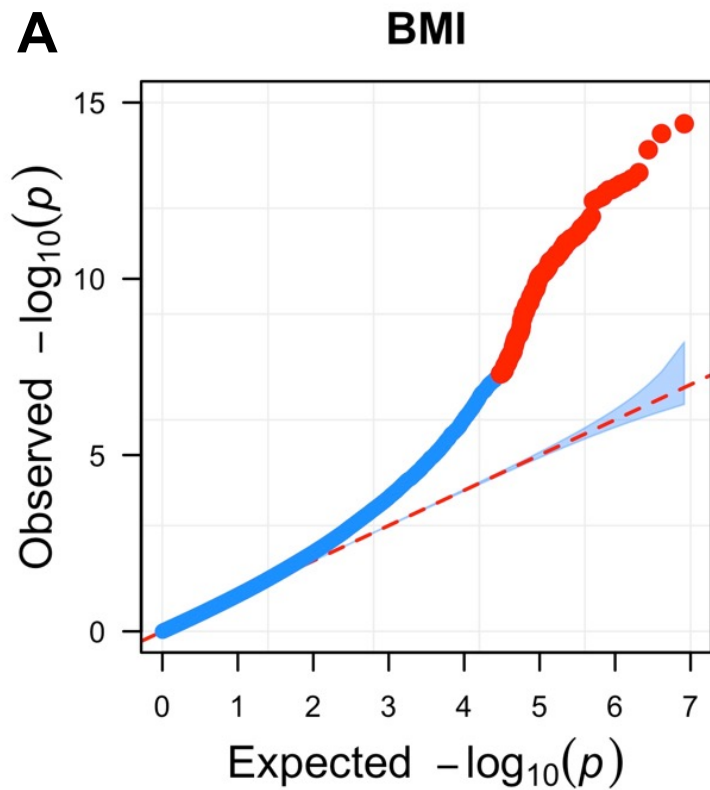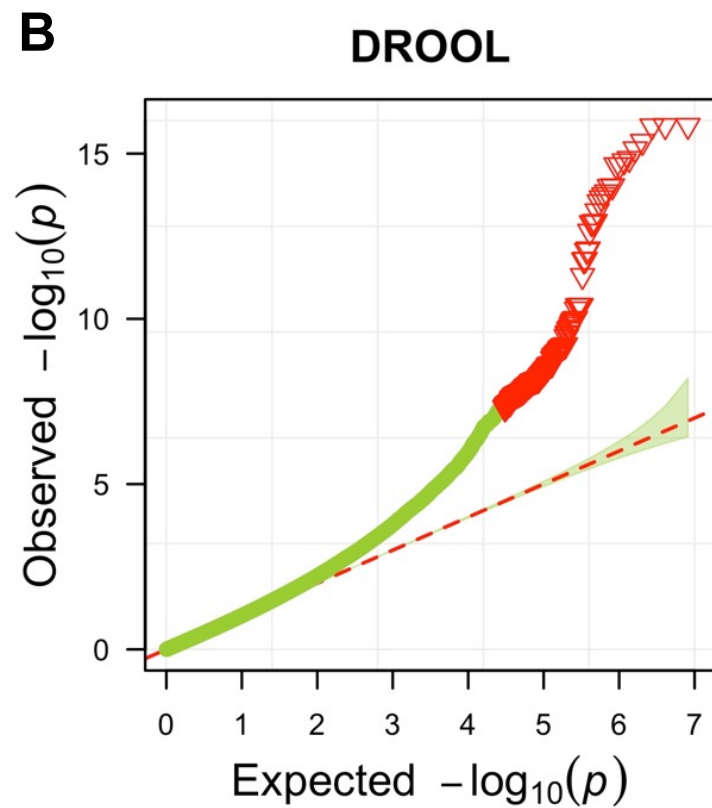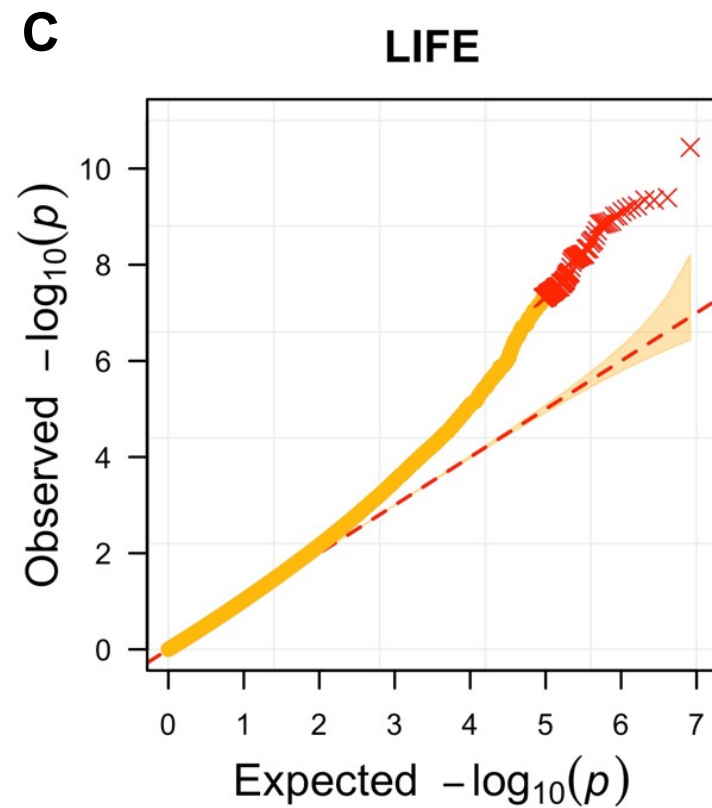

Supplement: Supplementary file 6 — Supplementary material [file mmc6.pdf]

**A**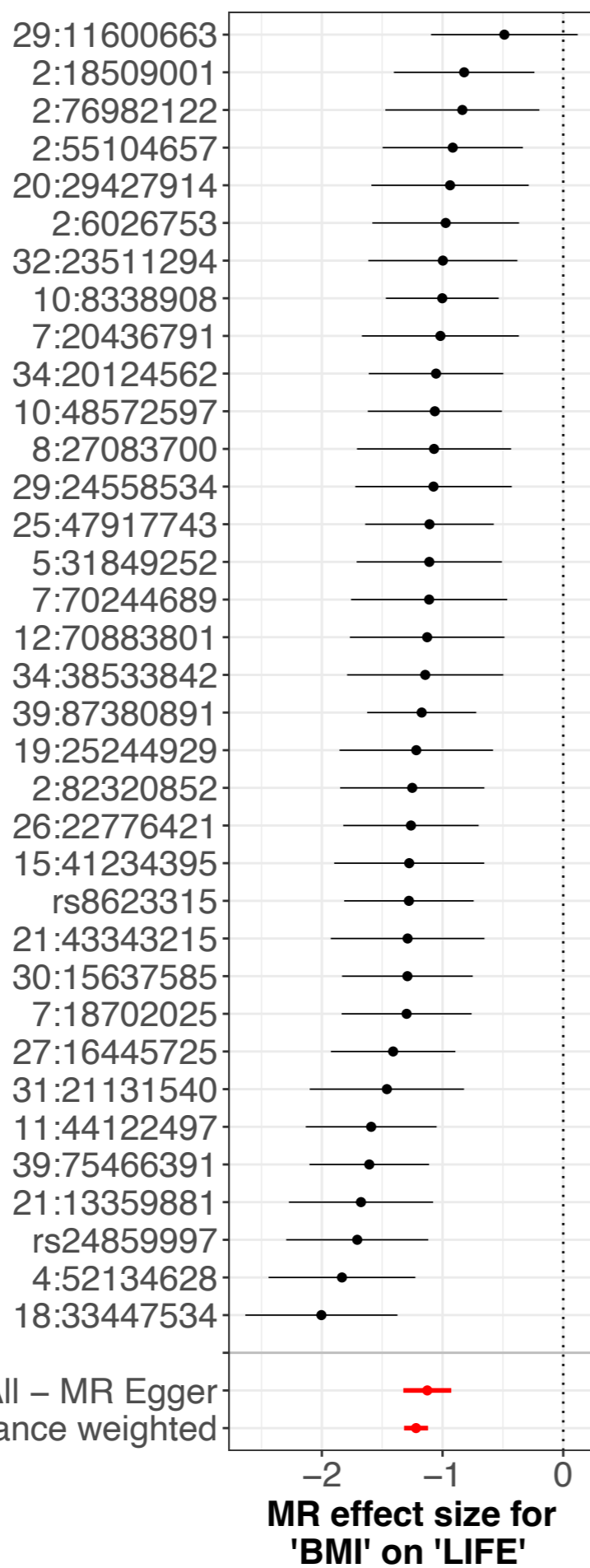**B**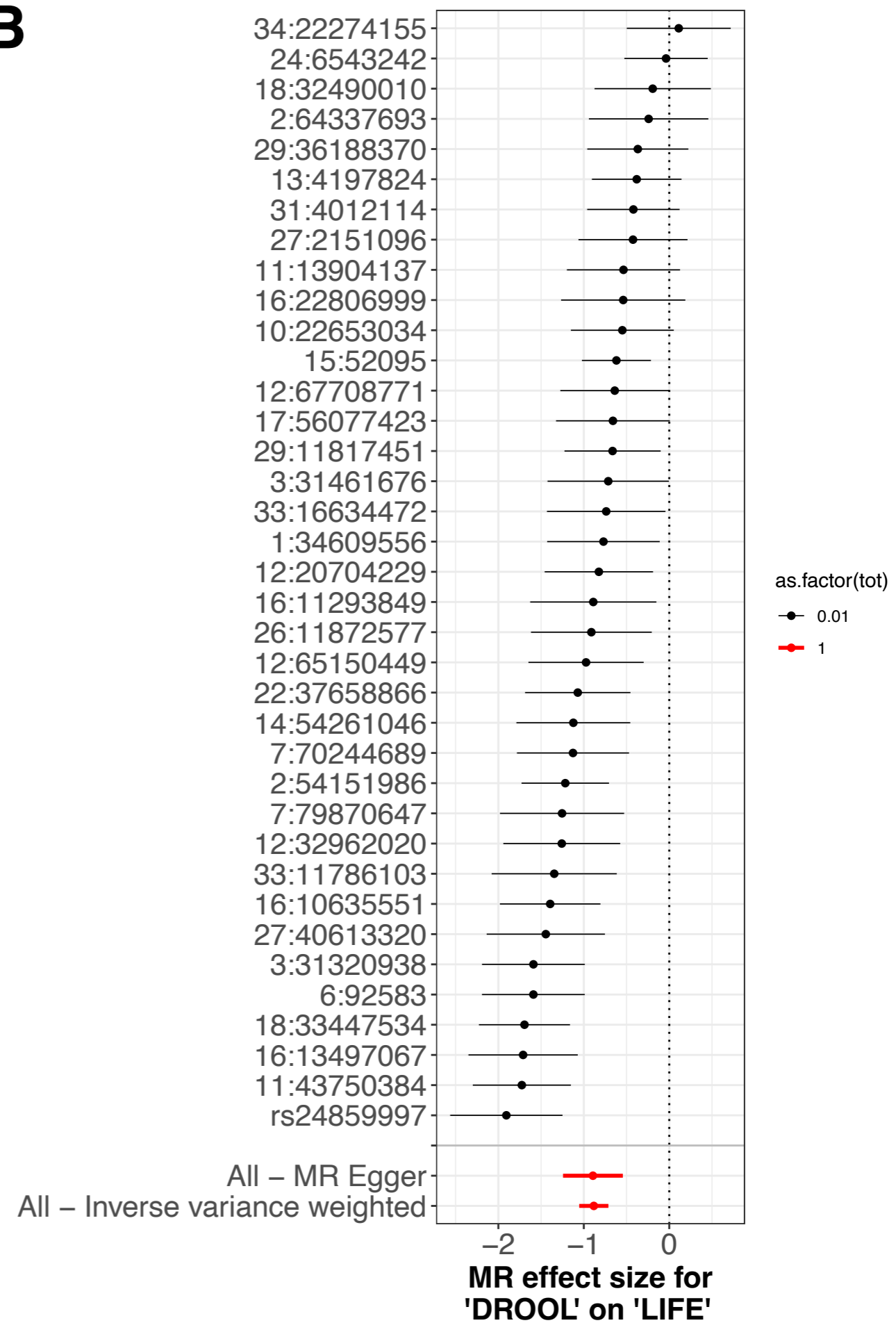

Supplement: Supplementary file 7 — Supplementary material [file mmc7.pdf]

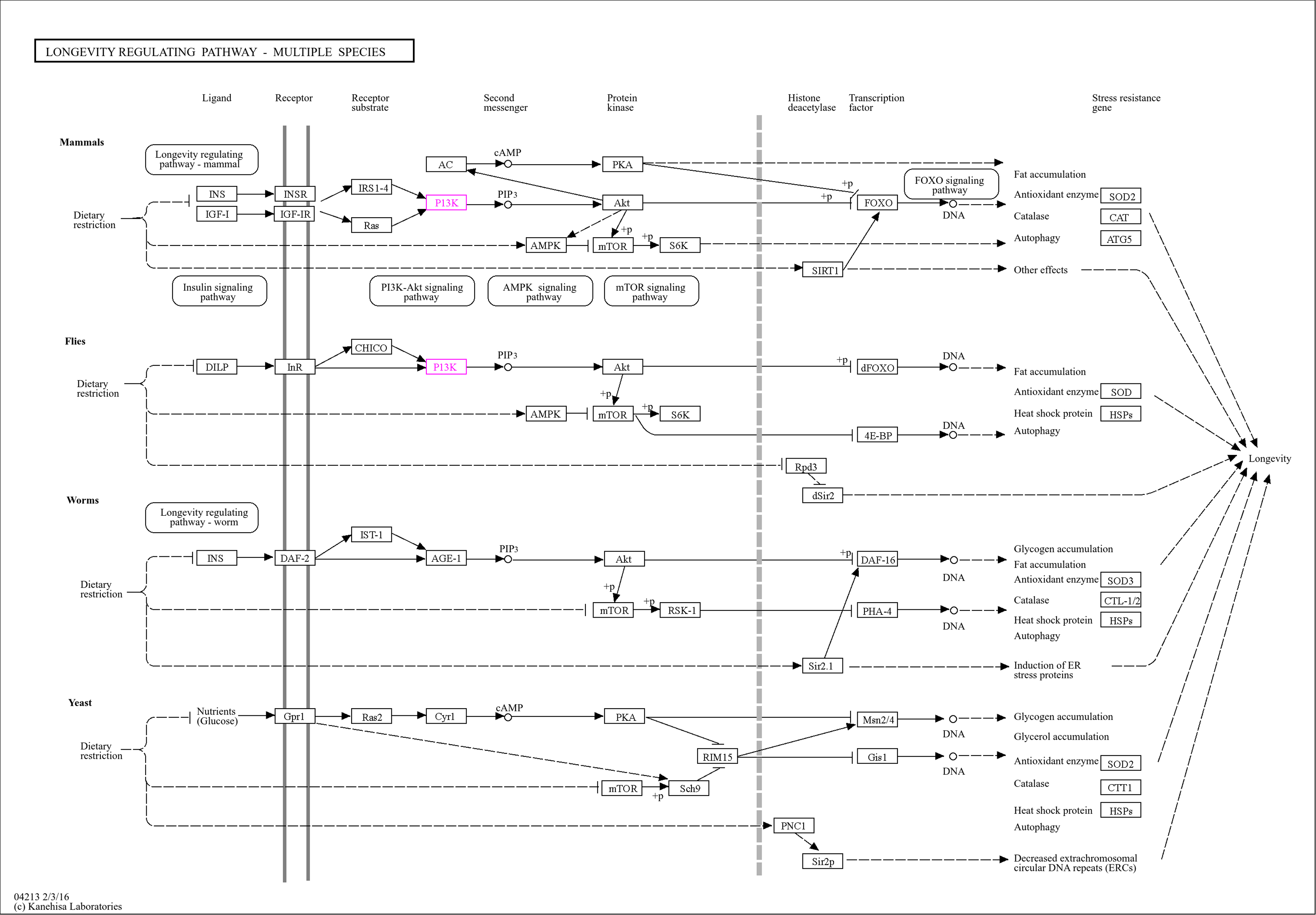

Supplement: Supplementary file 10 — Supplementary material [file mmc10.jpg]
