# Supplementary material for: Genome-wide statistical evidence elucidates candidate factors of life expectancy in dogs
Source: Mol Cells. 2024 Nov 22;48(1):100162. doi: 10.1016/j.mocell.2024.100162 (PMC11721540; doi:10.1016/j.mocell.2024.100162)
Supplement: Supplementary file 8 — Supplementary material [file mmc8.pdf]

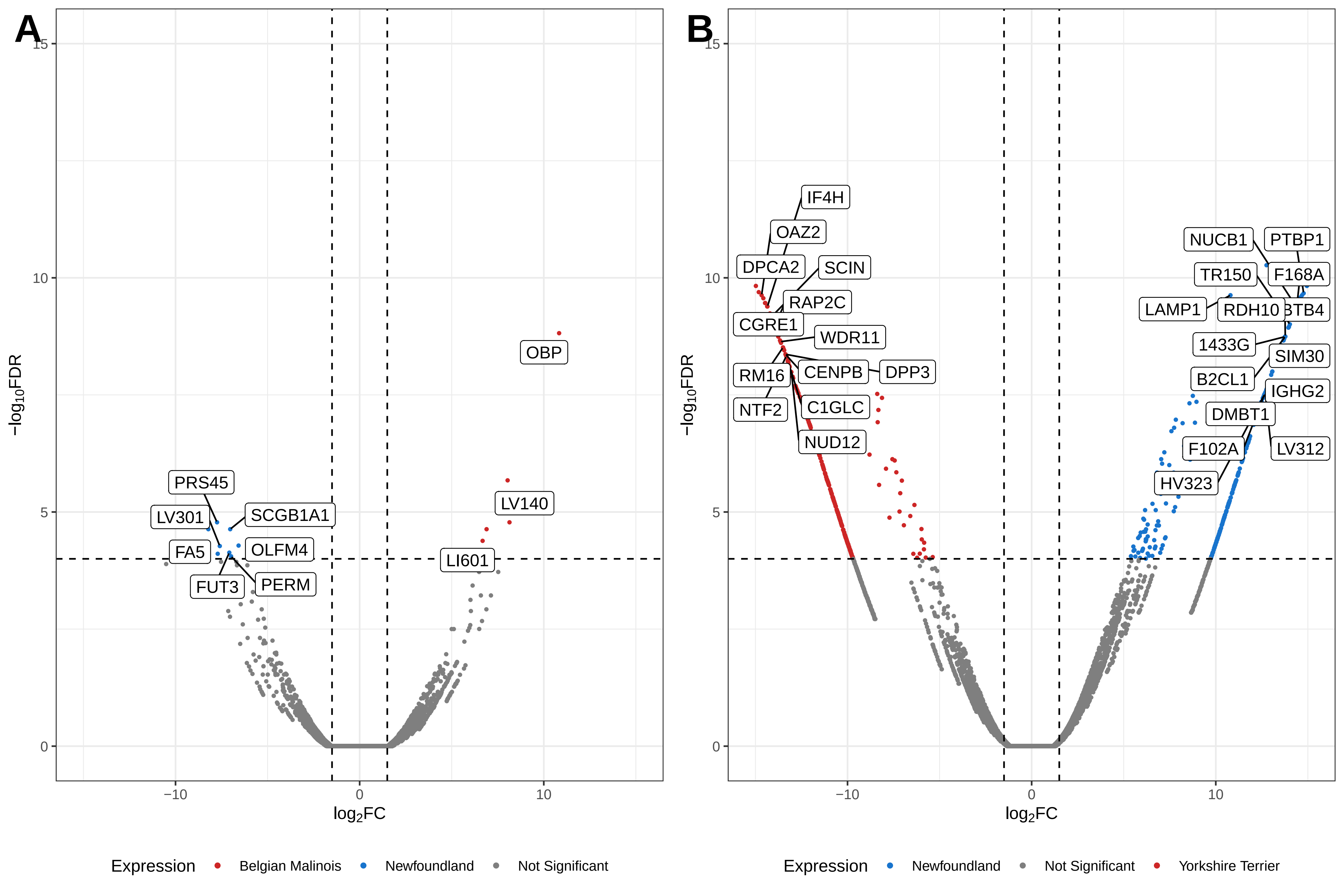
A
B
15
10
5
0
−10
0
10
−log10FDR
log2FC
PRS45
LV301
SCGB1A1
FA5
OLFM4
FUT3
PERM
OBP
LV140
LI601
IF4H
OAZ2
DPCA2
SCIN
RAP2C
CGRE1
WDR11
RM16
CENPB
DPP3
NTF2
C1GLC
NUD12
NUCB1
PTBP1
TR150
F168A
LAMP1
RDH10
BTB4
1433G
SIM30
B2CL1
IGHG2
DMBT1
F102A
LV312
HV323
Expression
Belgian Malinois
Newfoundland
Not Significant
Yorkshire Terrier
