# Supplementary material for: Genome-wide statistical evidence elucidates candidate factors of life expectancy in dogs
Source: Mol Cells. 2024 Nov 22;48(1):100162. doi: 10.1016/j.mocell.2024.100162 (PMC11721540; doi:10.1016/j.mocell.2024.100162)
Supplement: Supplementary file 9 — Supplementary material [file mmc9.docx]

**Supplementary Data Legends**

**Fig. S1. Diagnostic plots for model evaluation.** This presents diagnostic plots to evaluate the regression model predicting canine lifespan based on BMI and drooling. The posterior predictive check shows that model-predicted lines closely match the observed data distribution for lifespan. The linearity plot indicates that the residuals versus fitted values scatter around a flat line, suggesting the linearity assumption is met. The homogeneity of variance plot shows a consistent spread of residuals, indicating no major issues with homoscedasticity. The influential observations plot reveals that most points lie within the contour lines, suggesting no undue influence from individual data points. The collinearity assessment indicates low variance inflation factor (VIF) values for BMI and drooling, suggesting no multicollinearity concerns. The normality of residuals plot shows minor deviations but overall reasonable normality of residuals.

**Fig. S2. Phenotypes missingness visualization plot and the correlation plot.** The plot provides a visual summary of the relationships between various phenotypic traits in dogs. (A) The bar plot displays the percentage of missing values for various phenotypic traits in dogs. The x-axis represents the percentage of missing values, while the y-axis lists the phenotypic traits. Blue bars indicate the percentage of present data, and red bars indicate the percentage of missing data for each variable. (B) The correlogram depicts the pairwise correlation coefficients between the phenotypic traits. The traits are listed on both the x-axis and y-axis. The color and size of the circles represent the strength and direction of the correlations, with blue indicating positive correlations and red indicating negative correlations.

**Fig. S3. Box plot about drooling with BMI, and life expectancy.** Outliers are written next to the dots. A polyserial correlation formula is used for calculation. (A) A strong positive correlation is estimated between drooling and BMI. Outliers came out Cavalier King Charles Spaniels (1.24 BMI, drooling 2), and Tibetan Spaniels (0.70 BMI, drooling 2). (B) A strong negative correlation is detected between lifespan and drooling. The outlier came out to Scottish Deerhound (9.5 lifespan, drooling 1).

**Fig. S4. Distribution of the original drooling and the centered phenotype.** (A) Bar plot of unadjusted drooling factors of total individual of 722-dataset. (B) Bar plot of adjusted with the sex and body size to the drooling.

**Fig. S5. Quantile-Quantile plot (QQ plot) for all the GWAS results.** (A–C) 722-dataset QQ plot. Red dots indicate significant ps. The inflation factor (𝝀) values are 1.03, 1.02, and 1.03 respectively. BMI, drooling and lifespan respectively.

**Fig. S6. Forest plot of MR test.** The forest plot of the MR analysis for the both datasets. The forest plot is the result of the single SNP analysis in the MR. In the context of MR, a single SNP analysis involves using one genetic variant as the instrumental variable. Each line in the plot represents an individual SNP in both datasets. The horizontal axis shows the MR effect size, typically reflecting the change in the outcome variable per unit change in the exposure variable. These effect sizes are plotted as points along the axis, with lines representing confidence intervals. (A) BMI and lifespan at 722-dataset, (B) drooling and lifespan at 722-dataset.

**Fig. S7. Volcano plots of DEG analysis with dog breeds’ salivary gland RNA-seq data.** (A) Volcano plot displaying the differential gene expression between Belgian Malinois and Newfoundland. The x-axis represents the log2 fold change (log2FC) in gene expression between the two breeds, while the y-axis represents the -log10 false discovery rate (FDR). Genes with significant differential expression are highlighted and labeled. Red points indicate genes that are significantly upregulated in Belgian Malinois, and blue points indicate genes that are significantly upregulated in Newfoundland. All top genes were annotated. (B) Volcano plot of Newfoundland and Yorkshire Terrier and gene labeled top 15 signals. The full names of all comparisons of top genes are listed in the Table S7.

**Fig. S8. Longevity regulating pathway across multiple species.** The diagram illustrates the longevity-regulating pathways in mammals, flies, worms, and yeast, highlighting the conserved signaling mechanisms that influence lifespan across these species. Key components of these pathways include insulin signaling, PI3K-Akt signaling, and AMPK signaling, among others. In mammal, Insulin (INS) and *IGF1* interact with their receptors (INSR and IGF-1R) to activate IRS1-4 and Ras, leading to the activation of *PI3K* and subsequently Akt. This signaling cascade can activate mTOR or inhibit FOXO, impacting fat accumulation, antioxidant enzyme activity (SOD2, CAT), autophagy (ATG5), and other longevity-related processes. Dietary restriction can modulate these pathways, enhancing longevity by activating AMPK and deactivating mTOR signaling.

**Table S1. GWAS results summary.**

**Table S2. 722-dataset BMI GWAS results.**

**Table S3. 722-dataset drooling GWAS results.**

**Table S4. One-sample drooling and lifespan MR single SNP analysis result.**

**Table S5. One-sample BMI and lifespan MR single SNP analysis result.**

**Table S6. BUSCO results for the DEG analysis.**

**Table S7. Top enriched GO term analysis.**

**Table S8. GO term functional role annotation.**

**Appendix S1. Phenotype matrix.**

**Appendix S2. Phenotype matrix description.**

**Appendix S3. Phenotype comparison for the DEG-used breeds.**
